# Restrictions on contraceptive services for unmarried youth: a qualitative study of providers’ beliefs and attitudes in India

**DOI:** 10.1080/26410397.2022.2141965

**Published:** 2022-11-23

**Authors:** A Shukla, A Kumar, A Mozumdar, R Acharya, K Aruldas, N Saggurti

**Affiliations:** a Researcher, Research Institute for Medical and Health Sciences, University of Sharjah, Sharjah, United Arab Emirates.; b Senior Program Officer, Population Council, Delhi, India; c Senior Program Officer, Population Council, Delhi, India; d Senior Associate, Population Council, Delhi, India; e Implementation Science Coordinator, DeWorm3 Study, Christian Medical College, Vellore, India; f Director, Population Council, Delhi, India

**Keywords:** unmarried, youth, premarital sex, contraceptives, traditional belief, social dilemma

## Abstract

Sexual and reproductive health (SRH) of unmarried youth is an important issue, particularly in Indian society, where premarital sex is socially restricted. It is an uncomfortable subject for most people, including healthcare providers, who are responsible for catering to the reproductive health needs of youth. This is because of the prevailing social norms, where sex outside marriage is discouraged and stigmatised. These social norms give importance to virginity, and children outside marriage are not welcome. The present qualitative study was conducted in public health facilities (primary and secondary) to explore the attitudes of healthcare providers in providing contraceptive services to unmarried youth. In-depth interviews were conducted with family planning (FP) service providers (frontline healthcare workers [ASHAs] nurses and FP counsellors) between October 2017 and September 2018. Almost a quarter of the providers were either hesitant or against providing contraceptives to unmarried youth. Providers stated that they preferred emergency contraceptive pills for unmarried girls if they had already engaged in unprotected sex. Providers expressed strong personal views against premarital sex because they believed it was against existing social norms. Some providers were concerned about the possible negative reactions of the community if they recommended any contraceptive to unmarried youth. A few providers even considered it illegal to provide contraceptives to unmarried youth, though there is no such law in the country. Findings further indicated that though the country had launched programmes for improving adolescents and youth SRH, service providers were still conflicted between medical eligibility and social beliefs.

## Résumé

La santé sexuelle et reproductive (SSR) des jeunes célibataires est une question importante, en particulier dans la société indienne, où les rapports sexuels avant le mariage sont socialement restreints. C’est un sujet délicat pour la plupart des gens, y compris les agents de santé, à qui il incombe de répondre aux besoins de santé reproductive des jeunes. Cette situation s’explique par les normes sociales dominantes qui découragent et stigmatisent les rapports sexuels en dehors du mariage. Ces normes sociales donnent de l’importance à la virginité, et les enfants nés hors mariage ne sont pas les bienvenus. La présente étude qualitative a été menée dans des centres de santé publique (primaires et secondaires) pour explorer les attitudes des prestataires de soins de santé lorsqu’ils fournissent des services contraceptifs aux jeunes célibataires. Des entretiens approfondis ont été menés avec des prestataires de services de planification familiale (agents de santé de première ligne (ASHA), infirmières et conseillers en planification familiale) entre octobre 2017 et septembre 2018. Près d’un quart des prestataires étaient réticents ou opposés à l’idée de donner des contraceptifs à de jeunes célibataires. Les prestataires ont déclaré qu’ils préféraient les pilules contraceptives d’urgence pour les jeunes filles célibataires si elles avaient déjà eu des rapports sexuels non protégés. Ils ont exprimé des idées personnelles fortes contre les rapports sexuels avant le mariage car ils pensaient qu’ils étaient contraires aux normes sociales existantes. Certains prestataires s’inquiétaient des réactions négatives possibles de la communauté s’ils avaient recommandé un contraceptif à de jeunes célibataires. Quelques prestataires considéraient même qu’il était illégal de fournir des contraceptifs à de jeunes célibataires, même s’il n’existe pas de telle loi dans le pays. Les résultats ont également révélé que, même si le pays a lancé des programmes pour améliorer la SSR des adolescents et des jeunes, les prestataires de services étaient encore partagés entre l’éligibilité médicale et les croyances sociales.

## Resumen

La salud sexual y reproductiva (SSR) de personas jóvenes solteras es un asunto importante, en particular en la sociedad india, donde el sexo prematrimonial es restringido socialmente. Es un tema delicado para la mayoría de las personas, incluidos los prestadores de servicios de salud, que son responsables de atender las necesidades de salud reproductiva de la juventud. Esto se debe a las normas sociales predominantes, donde el sexo extramatrimonial es desaconsejado y estigmatizado. Estas normas sociales dan importancia a la virginidad y los hijos nacidos fuera del matrimonio no son bienvenidos. El presente estudio cualitativo fue realizado en unidades de salud pública (de atención primaria y secundaria) para examinar las actitudes de prestadores de servicios de salud al proporcionar servicios de anticoncepción a personas jóvenes solteras. Se realizaron entrevistas a profundidad con prestadores de servicios de planificación familiar (PF) (trabajadores de salud de primera línea {ASHA}, enfermeras y consejeros en PF), entre octubre de 2017 y septiembre de 2018. Casi una cuarta parte de los prestadores de servicios eran renuentes o estaban en contra de proporcionar anticonceptivos a personas jóvenes solteras. Los prestadores de servicios decían que preferían las píldoras anticonceptivas de emergencia para las niñas solteras si ya habían tenido sexo sin protección; además, expresaron firmes puntos de vista personales en contra del sexo prematrimonial porque creían que iba en contra de las normas sociales vigentes. Algunos prestadores de servicios se preocupaban por las posibles reacciones negativas de la comunidad si recomendaban un método anticonceptivo a una persona joven soltera. Algunos prestadores de servicios incluso creían que era ilegal proporcionar anticonceptivos a personas jóvenes solteras, aunque no existe tal ley en el país. Los hallazgos indican que, aunque el país había lanzado programas para mejorar la SSR de adolescentes y jóvenes, los prestadores de servicios aún sentían conflicto entre la elegibilidad médica y las creencias sociales.

## Introduction

In many Asian cultures, including India, premarital sex is considered taboo because a lot of importance is given to virginity.^1^ Also, procreation is believed to be the only reason for a sexual relationship and thus is not encouraged before marriage. Girls have to face greater stigma due to the possibility of becoming pregnant, a state they cannot hide from the community. Although premarital sex is not socially accepted in many settings, there is emerging evidence of sexual activity among unmarried youth.^2–4^ With an increase in age at first marriage, increased exposure to media and the increasing opportunities to meet people of the opposite sex in academic, vocational and work-related environments, youth's involvement in premarital sexual activity has also increased.^5^

This increase in premarital sex highlights the need and necessity to provide sexual and reproductive health (SRH) related services to all youth, irrespective of their marital status. Studies from India show that without proper information and services, 2–30% of unmarried sexually active young women experience an unplanned pregnancy and abortion.^6,7^ Moreover, the risks of sexually transmitted infections (STIs) and unplanned pregnancy were higher among people below the age of 25 years as they often engaged in unsafe sex, were involved with multiple partners, in contact with sex workers, and often did not use contraceptives or used them ineffectively.^2,8^ Evidence from Asia suggests that although the proportion of men involved in unsafe sexual practices was higher than women, a significant percentage of women too were involved in unsafe sexual practices, such as having multiple partners at a young age and low use of condoms.^9,10^

A study from India reported that unmarried women were more likely to be forced into non-consensual sexual relations, leading to unwanted pregnancies and abortions in the second trimester.^11^ A similar study in China found that almost half of the pregnancies among unmarried girls ended up in abortion. Many of those were unsafe and posed a greater threat to their life and well-being.^4^ Unmarried women also have to suffer issues that include timely pregnancy identification, not having a say in decision-making, and confidentiality in selecting an abortion facility.^11^

For adolescents in developing countries, very little comprehensive information regarding access to contraceptives and abortion services is available.^12^ Several studies have recognised provider bias, i.e. provider’s self-imposed restrictions in providing contraceptive services, as an important barrier to women’s sexual and reproductive rights.^13^ Studies have also found that access to SRH services, including contraceptive services, is quite different for married compared to unmarried youth. Unmarried youth face additional constraints in accessing contraceptive services and related information due to existing social norms that discourage and stigmatise premarital sex. Due to prevailing social norms for premarital sex, healthcare providers often feel uncomfortable discussing sexual issues with unmarried young people.^14^ A study in Senegal reported that even when there were no legal restrictions, providers were reluctant to provide oral contraceptive pills to unmarried young women and advised them against premarital sex.^15^ In Nigeria, healthcare providers perceived the provision of contraceptives for unmarried adolescents as promoting sexual promiscuity.^16^ There is evidence of how providers’ negative attitudes towards premarital sex increased difficulties for unmarried youth accessing and utilising contraceptive services.^17,18^

Although universal access to SRH services among adolescents has been on the global family planning (FP) agenda for a long time, it was recognised at the 2017 Family Planning Summit that progress had been slow and inconsistent.^19^ Sexual and reproductive rights are enshrined in many international conventions, agreements, laws and declarations; yet, young people around the world face challenges in exercising their sexual and reproductive rights. Access to non-stigmatising and non-judgmental sexual and reproductive healthcare services for the young population is important to upholding their sexual and health rights.

Following the global call, the Indian government took several initiatives to cater to the needs of adolescents. The Adolescent Reproductive and Sexual Health (ARSH) strategy was formulated to improve awareness and access to reproductive health services among married and unmarried youth.^20^ National data from India indicated that the proportion of unmarried young girls aged 15–24 who were involved in premarital sex increased from less than 1% in 2005–2006 to about 2.3% in 2015–2016. On the other hand, the proportion of women currently using modern contraceptives among unmarried women aged 15–24 who ever had sex was 9% as compared to currently married women of the same age group (21%) in 2015–2016.^21,22^ A study in India reported that young unmarried rural men had limited knowledge about preventing pregnancies. Though a majority of them knew about condoms, they saw them more as a preventive measure against STIs than to avoid unwanted pregnancies.^23^ The study also pointed out that these men were curious for more information but felt ignored by healthcare providers. The abovementioned facts indicate the gaps in knowledge and access to SRH service among unmarried youth in the country, and this makes access to sexual and reproductive health and rights (SRHR) of young people, irrespective of their marital status, an extremely crucial issue to be discussed.

However, research on providers’ behaviour and preferences in India in order to understand barriers in accessing the supply of contraceptives has largely focused on married women. Low contraceptive use among unmarried youth calls for understanding barriers they also face in accessing contraceptives. The present paper explores providers’ beliefs, preferences, and attitudes towards contraceptive service provision among unmarried youth in two large north Indian states.

## Materials and methods

### Data collection

This qualitative study is part of a larger study conducted to assess providers’ attitudes and preferences in providing FP services. However, this paper is only based on data related to the provision of contraceptive services for unmarried youth. The study was conducted in four selected districts of Bihar and Uttar Pradesh (UP). The two study districts from each state were selected to represent both good and poor-performing areas in the context of FP: one district with modern contraceptive use higher than the state average and with a balanced method mix, and another district with modern contraceptive use lower than the state average and with a skewed method mix (where the overall contraceptive prevalence in an area is dominated by one particular method).^24^ From each district, two administrative blocks were selected based on the ratio of the intrauterine contraceptive device (IUCD) to female sterilisation services provided in the public health facilities in the block (one block with a high ratio and one with a low ratio). Data for this study were collected between October 2017 and September 2018 from FP service providers engaged in public health facilities – from each district, one district hospital, two community health centres or primary health centres and two sub-centres (see Appendix). One of each facility from two administrative blocks was selected. The study team conducted 60 interviews of public health providers: accredited social health activists (ASHAs)* (*N* = 16), staff nurses (SNs)† or auxiliary nurse midwives (ANMs)‡ (*N* = 24) and FP counsellors (FCs)§ (*N* = 20) (a detailed distribution of providers by type of facility is given in Appendix). Field investigators who interviewed study participants possessed graduate degrees and had experience conducting qualitative interviews. Investigators were trained by key research persons on the study design, interview guidelines and the process of conducting qualitative interviews.

Data were collected through in-depth interviews with healthcare providers using a guideline relevant to the study’s objectives. Providers were asked about their views on providing contraceptive services and information to unmarried adolescents and youth less than 25 years of age and their reasons for those views. Questions included: (1) what methods the provider preferred for unmarried youth and (2) what they offered if an unmarried boy or girl approached them for information or contraceptive methods.

All interviews were conducted at health facilities in the providers’ native language, i.e. Hindi, and it took an average of forty minutes to complete one interview. All interviews were audio-recorded after receiving consent to participate and record the interviews. The recorded interviews were transcribed and analysed. Details about study design, data collection, and analytical methodology are published in another article.^25^

### Analysis

The analysis for this paper followed the grounded theory approach. The starting point for this grounded theory was to assess the providers’ attitudes and preferences in providing contraceptives to unmarried youth. The primary objective of this study was to understand the providers’ preferences and explore the key reasons associated with them. Interviews were transcribed verbatim in Hindi by field investigators. The research team thoroughly reviewed the transcripts and developed an initial code list. The code list was then discussed and finalised, and codes were grouped into broad categories based on emerging patterns. The authors coded and analyzed the final transcripts using ATLAS.ti software and summarised the results. Later, the authors translated relevant verbatim transcripts into English and included them in this paper. A reverse translation was conducted to ensure that the original meaning of the Hindi transcripts did not change. The authors listened to the audio recording of all interviews to check the quality and validity of the transcripts. A summary of providers’ interviews is included in this paper using illustrative quotes; all references to individuals by name within the quotes have been changed to protect their identity.

### Ethical considerations

The Institutional Review Board (IRB) of the Population Council, New York, USA, approved the ethical considerations of this study on 10 July 2017. This project was also approved by the ethical committees of the Ministry of Health and Family Welfare, the Government of India, and the state governments of Bihar (18 September 2017) and UP (19 March 2018). In this study, each participant provided informed consent in writing, confirming their voluntary participation in the study. Confidentiality and privacy of the information were maintained throughout the study and report writing by using assigned codes to identify participants instead of personal information and identity.

## Results

**Figure 1.**
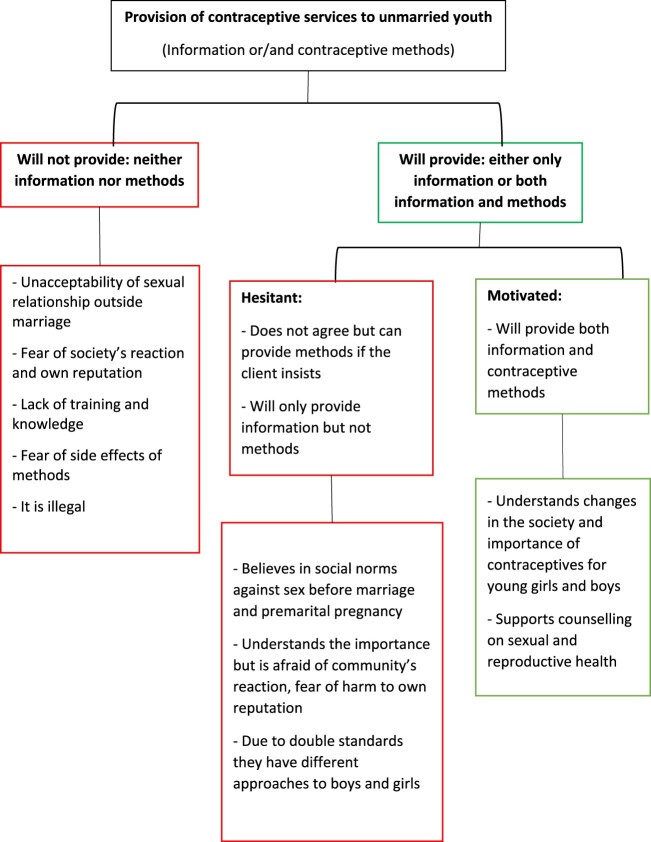
Public health providers’ perception of the provision of contraceptive information and methods to unmarried youth and the reasons for it

After the initial analysis of the data, the research team classified providers into three groups based on their attitudes and preferences regarding contraceptive services to unmarried youth. The first group consisted of providers who were supportive and motivated, i.e. providers who reported being open to providing counselling and offering contraceptives to unmarried youth without any hesitation. The second group comprised hesitant providers, i.e. providers who showed some hesitation to offer contraceptives to unmarried youth. Providers in the third group were those who reported that they would never provide contraceptive services to unmarried youths. Figure 1 shows the groups of providers by their attitudes and beliefs and a summary of the reasons they shared with the research team. This paper discusses these three abovementioned categories of providers, their perceptions of access to contraceptive services for unmarried youth and the reasons for their perceptions.

### Supportive and motivated providers

Of the 60 providers interviewed, 32 (four ASHAs, 10 nurses and 18 counsellors) reported that they would provide contraceptive methods to unmarried young girls and boys without hesitation. These providers preferred offering emergency contraceptive pills (ECPs) and condoms to unmarried youths, and some were willing to give oral contraceptive pills too. The providers preferred condoms as they can help safeguard against STIs and have no side effects. Also, they reported that unmarried youth mostly demanded ECPs, to avoid visiting health facilities and making their sexual activities known in public.

*“I will give condoms as it will keep them protected from both STIs and pregnancy.”* (Nurse, Bihar, ID2)

These providers acknowledged that society was changing, with more and more people engaging in premarital sexual relationships. For this reason, these providers believed that youth should be given information and methods, as these would protect them from STIs, unintended pregnancies and resultant abortions. They further expressed that even if the boy or girl was not sexually active, they could use the information in the future after marriage.

*“Unmarried girls need contraceptive methods and related information because if they conceive, society will not let them live. Sometimes they resort to abortions. We deal with such cases all the time. Recently one young man came to terminate a 7-month pregnancy of his girlfriend. The girl did not seem ok and was mentioning pain in the abdomen. She was only 19 years old, and we transferred her to the district hospital.”* (Nurse, UP, ID65)

Some providers felt it was their responsibility to provide information and counselling to unmarried youth. These providers expressed that adolescents should be allowed to take their own decisions, and healthcare providers should provide them with contraceptive services without judgment and restrictions based on age-old social norms. The world has changed, the proportion of sexually active young girls and boys has increased, and hence it is necessary to provide them with complete information about contraceptives. Providers also reported that in the absence of complete information and access to contraceptive methods, there was a risk that these young girls and boys would resort to unsafe abortion methods.

“*I will provide information and contraceptive methods [to unmarried girls and boys]. If they get involved in unsafe sex, that would be more harmful. That is why I give them condoms or other methods they ask.”* (FP Counsellor, Bihar, ID81)

“*It is necessary to provide complete information about available methods and side effects. Otherwise, they will go to the medical shop and may use any harmful medicines. We have been trained to provide methods to unmarried [girls and boys]; otherwise, they will resort to abortion and face complications. We can give them ECPs and condoms.”* (Nurse, UP, ID76)

#### Specific training catered around adolescents and young unmarried population

One provider mentioned how ARSH training helped in overcoming her prejudices.

“*Earlier, we did not give them contraceptives, but after ARSH training, we have been taught to give them too. The pills also help them [to regulate their] menstruation cycle. I will give them a method to avoid unwanted pregnancy.”* (Nurse, Bihar, ID72)

Three providers mentioned that they referred some unmarried youth to ARSH counsellors for in-depth counselling.

### Hesitant providers

Ten providers showed varying degrees and types of hesitancy in providing contraceptive services to young unmarried clients. For example, three providers reported that they would provide information about contraceptives to unmarried youth as knowledge is necessary; however, they will not provide or recommend any contraceptive methods to them. Seven providers agreed to offer the methods with some hesitation and only under certain circumstances. For example, they were willing to provide a girl with ECP if she had unprotected sex and feared that she would conceive. Otherwise, they would provide condoms only to boys to avoid STIs or provide contraceptives if a boy/girl insisted.

“*I will scold them, and if she still insists, I will throw a condom at them. It is a sin to be involved in such activities, but if she begs [for contraceptives], I will give her [condoms].”* (ASHA, Bihar, ID101)

#### Reasons for hesitancy in providing contraceptive services to unmarried youth

##### Social norms against premarital sex and pregnancy

Despite their disapproval of premarital sex and contraceptives, some providers reported that they would provide contraceptive methods to prevent girls from conceiving before marriage. This was because such pregnancies were not accepted in the community, and such a girl would be ridiculed for it by her family and community members.

“*First, I will scold them for such actions; it is a sin to be involved in sexual activities before marriage. But if the girl requests and is at the risk of pregnancy, I will unwillingly give her ECPs.”* (ASHA, Bihar, ID101)

##### Fear of society’s reaction and damage to one’s reputation

A few providers were also concerned about their reputation and reported that they were only comfortable providing methods to known people and those who would not be promiscuous. They also reported that they feared the community's reaction if community members found out they provided contraceptives to unmarried youth.

“*I will give method only if they ask. Here in this village, people will have an objection to it. But if someone is in need, I will provide unwillingly.”* (ASHA, Meerut, ID10)

##### Sexual double standards

One provider said that she had been taught to provide contraceptives only to boys and not girls.

“*We have been taught to provide condoms to boys to save them from sexually transmitted diseases, but we have not been told to provide anything to girls.”* (ASHA, Bihar, ID99)

In reality, no government guideline instructs providers to talk only to boys or provide condoms only to boys. This indicates the presence of the provider’s self-imposed gender bias in counselling and providing contraceptive services and is a sign of gender discrimination.

### Providers who do not want to offer any contraceptive services to unmarried youth

Fourteen providers said they would not recommend contraceptives to unmarried girls and boys under any circumstances. Among these providers were five ASHAs, eight nurses and one FP counsellor.

“*It is not right to give [contraceptives] to them [unmarried boys/girls], and [therefore] I will not give it to them.”* (Nurse, Bihar, ID67)

#### Reasons for not providing contraceptive services to unmarried youth

##### Social norms against sex before marriage

Providers reported that it was against the norms of society for girls and boys to have sex before marriage. These providers shared that they discouraged unmarried boys and girls from participating in sexual activities.

“*I tell unmarried girls and boys not to indulge in sexual relationships before marriage. I will not give any contraceptive method to them.”* (ASHA, Bihar, ID16)

##### Fear of society’s reaction and damage to one’s reputation

Two providers mentioned that they did not want to provide contraceptives to unmarried youth because they expected hostile reactions from their families and the community.

“*I will not give. If their parents come to know this, they will fight with me.”* (ASHA, UP, ID11)

“*I will not give [methods to unmarried youth]. If their parents come to know this, they will attack me.”* (ASHA, Barabanki, ID15)

##### Fear of sexually transmitted diseases caused by premarital sex and possible side effects of contraceptive use

Providers mentioned that premarital sex might lead to STIs; therefore, unmarried girls and boys should not indulge in premarital sex and should wait until marriage. Some providers are worried about the side effects of contraceptives when used at a young age. Providers believed contraceptives could harm the uterus or cause infertility in the future.

“*I will not give [contraceptives] to them. Why should I get involved in all this? They should not be in relationships before marriage. It's not good for their health and can damage their uterus too.”* (Nurse, Bihar, ID69)

##### Lack of knowledge and training

Some responses from the providers indicated their lack of training for counselling unmarried youth on sex and contraceptive use. One provider mentioned that she did not provide contraceptive methods to unmarried youth because she had never been told to do so.

“*I will not give [contraceptives] to unmarried youth in any case … neither I have been told to do so [give contraceptives to unmarried youth], nor I will give.”* (ASHA, UP, ID5)

One nurse even feared that it was illegal to provide contraceptive services to those who were not married.

“*It is against the law to give them methods. If someone finds out that they [unmarried clients] have a method, they [boy/girl] will not tell their part, and I will be blamed for everything.”* (Nurse, UP, ID65)

## Discussion

In the present study, providers acknowledged that the sexual behaviour of young Indians is changing as more and more young individuals are getting involved in premarital sex. Some providers mentioned that they were often approached by unmarried youth for contraceptives. Half of the providers in the present study were either hesitant to provide contraceptive information/methods or categorically said they would not provide contraceptive methods to unmarried youth. These providers mostly cited moral and social norms as a reason for not complying with the medical eligibility of unmarried youth. Despite increased demand for contraceptives among unmarried youth and the government programme allowing information dissemination and services to youth irrespective of their marital status, providers were imposing restrictions, citing social norms disapproving premarital sexual activity.

Both types of providers – for or against contraceptive use among unmarried youth – wanted to prevent unsafe sex among unmarried youth. Those who did not favour contraceptive use wanted to prevent unsafe sex by imposing restrictions on premarital sex instead of promoting contraceptive use. Similar findings were reported in studies from Senegal, Nigeria and Ethiopia, where healthcare providers tended to promote abstinence rather than providing contraceptive services to unmarried young populations.^14–16^ Many studies have recognised provider bias: that is, providers’ self-imposed restrictions in order that young, unmarried people should not indulge in premarital sex.^26^ These restrictions from providers do not comply with medical eligibility and are certainly against the clients’ rights. Though the group of hesitant providers acknowledged that using contraceptives like condoms could save unmarried youth from STIs and unintended pregnancies, they continued to restrain themselves due to fear of negative repercussions from the community for distributing contraceptives.

Irrespective of medical eligibility, providers had personal views on which methods unmarried youths should use. Providers preferred only certain contraceptives (e.g. condoms) due to inadequate information/training. These providers feared that methods such as IUDs and oral contraceptive pills could cause side effects and infertility among women. Healthcare providers have even cited similar reservations for young married women who did not yet have children.^25^ Emergency contraception was the most preferred method and was suggested as a last resort to prevent unmarried girls who had unprotected sex from conceiving and thus from being shamed by the community due to an unintended pregnancy before marriage. This showed that the providers were willing to help sexually active girls to avoid pregnancies but were not willing to supply regular pre-emptive methods.

A positive finding was that about half of the interviewed providers reported they would give information and contraceptive methods to unmarried youth. These providers recognised the changes in social settings. They agreed that providing information and methods to young populations was necessary to prevent unsafe sex that could lead to STIs, unwanted pregnancies and abortion of unplanned pregnancies.

In response to the global call for preventing early pregnancy and poor reproductive outcomes among adolescents, India removed legal barriers to accessing SRH services for unmarried adolescents.^27^ Currently, FP services are being provided in India under the Reproductive Maternal Newborn Child and Adolescent Health (RMNCH + A) Program. However, it has been found that even if services existed, social norms posed considerable barriers for young people who needed access.^23,25,28^ Understanding the sensitivity of the matter, the Government of India is focusing on training providers on the subject. It has allotted counsellors specifically to handle these matters in all public facilities. The recent initiative by the Government of India to designate one ARSH counsellor per facility and provide training on ARSH has shown positive impacts. Providers accepted that the training had sensitised them towards these issues and helped them overcome prejudices. However, during field visits for the present study, the research team observed that the availability of these counsellors was not universal in health facilities across the two study states.

The Beyond Bias Project focused on ensuring young people’s access to non-judgmental, quality counselling and provision of contraceptive services. It recommended an adaptable behaviour change model and design principles for shifting provider-bias across diverse contexts.^26^ The Project promoted behaviour change mechanisms for providers, that (i) humanise bias and hold up a mirror for providers, (ii) improve emotional connectivity with youth and (iii) address providers’ fears of community backlash. In the present study too, providers cited social norms and the community’s reaction as reasons for not providing contraceptive services to unmarried youth. A tailored training guideline based on the recommendations of the Beyond Bias Project might help curb bias and prevent unwanted pregnancies and their consequences.

## Limitations

The present findings provide the basis to further conduct well-constructed research. The current sample did not include doctors from public health facilities. Within the Indian public health sector, most contraceptive counselling is provided by cadres of healthcare workers at the grassroots level, not by medical doctors. This is being done to reduce the workload of the already overburdened doctors. Therefore, the findings can be considered representative of the overall situation of contraceptive counselling and services available for unmarried youth in India, especially at public health facilities in North India.

## Conclusion

The findings of this study reflect that providers’ hesitance, combined with strong prevailing social norms against premarital sex, prohibits them from providing contraceptive services to unmarried youth. This indicates how the sexual health of unmarried youth is still stuck between social norms and medical ethics, while it is well articulated that addressing the contraceptive needs of young girls is critical in preventing premarital pregnancy, which may result in abortion and subsequent reproductive illnesses. The provision of information and meeting the contraceptive needs of unmarried youth may ensure that their reproductive rights and well-being are upheld. The findings of this study suggest that appropriate training of healthcare providers will help them to overcome their self-imposed restrictions and to manage prevailing social norms against providing contraceptive services to unmarried youth. Additionally, further research is necessary to find new ways to reduce providers’ moral conflict between social and public health-related issues in reaching out to young, unmarried girls and boys.

## Data Availability

Data that supports the findings of this study are available upon reasonable request to the authors.

## Footnotes

* Accredited social health activists (ASHAs) are community health workers who create awareness on health, counsel women on health and contraceptives, mobilize the community and facilitate access to health related services available at the village/sub-centre/primary health centres. They also provide a minimum package of curative care for minor ailments such as diarrhoea and fevers and distribute condoms and contraceptive pills.

† Staff nurses have graduate or diploma degrees in nursing and their roles and responsibilities involve first-aid provision, drug administration, critical care for patients, assisting doctors in OTs and maintaining records.

‡ Auxiliary nurse midwives (ANMs) are village-level female health workers in India who work at village health sub-centres. Their work includes providing maternal and child health services along with family planning services.

§ Family Planning Counsellors are placed at the public sector health facilities in India. They play a key role in increasing awareness on sexual and reproductive health and generating demand for the various family planning services provided at the facilities.

## Appendix

**Table A1.** Distribution of type of providers interviewed by type of facilities

	ASHA	Nurse/ANMs	FP Counsellor	Total
District hospital	NA	8	9	17
Community health centre	NA	9	10	19
Primary health centre	NA	7	1	8
SC	16	NA	A	16
Total	16	24	20	60
